# A Novel Mobile Phone App (OncoFood) to Record and Optimize the Dietary Behavior of Oncologic Patients: Pilot Study

**DOI:** 10.2196/10703

**Published:** 2018-11-20

**Authors:** Till Orlemann, Dejan Reljic, Björn Zenker, Julia Meyer, Bjoern Eskofier, Jana Thiemt, Hans Joachim Herrmann, Markus Friedrich Neurath, Yurdagül Zopf

**Affiliations:** 1 Hector Center for Nutrition, Exercise, and Sports Department of Internal Medicine 1 University Hospital Erlangen, Friedrich-Alexander University Erlangen-Nürnberg Erlangen Germany; 2 Embedded Systems Institute Department of Informatics Friedrich-Alexander University Erlangen-Nürnberg Erlangen Germany; 3 Machine Learning and Data Analytics Lab Department of Computer Science Friedrich-Alexander University Erlangen-Nürnberg Erlangen Germany; 4 Department of Internal Medicine 1 University Hospital Erlangen, Friedrich-Alexander University Erlangen-Nürnberg Erlangen Germany

**Keywords:** cancer, mobile apps, diet, nutrition, protein intake, smartphone, mobile phone

## Abstract

**Background:**

Catabolism and tumor-specific therapy lead to reduced nutrient intake and weight loss in cancer patients. Maintaining a specific individualized diet can be challenging for the patient as the nutritional counseling options are limited. Monitoring of nutrient intake and frequent feedback are, however, vital for successful nutritional therapy because they support the patient’s compliance and realization of dietary therapeutic goals.

**Objective:**

This study aimed at investigating the feasibility and applicability of a novel mobile phone app to assess and evaluate dietary behaviors in oncologic patients.

**Methods:**

To determine dietary habits and food preferences in oncologic patients, initially 1400 nutritional records were evaluated and analyzed. The results provided the basis for creating a nutritional mobile phone app. Key requirements for the app included simple handling, recording the daily intake, and a comparison of nutrient targets and current status. In total, 39 cancer patients were recruited for the study; 15 patients dropped out prior to the study. All patients received a nutritional anamnesis, nutritional analysis, and nutritional counseling. Individual energy and nutrient aims were defined. The intervention group (n=12) additionally used the app. Weight and body composition of each group were evaluated after 4 weeks.

**Results:**

The app group gained significantly more weight (*P*=.045; mean weight 1.03 kg vs –1.46 kg). Also, skeletal muscle mass showed a significant increase in the app group (*P*=.009; mean skeletal muscle mass 0.58 kg vs –0.61 kg) compared with the control group. There was no significant difference between groups relating to the daily protein intake (*P*=.06). Additionally, there was a decrease in macronutrient intake during the study period in the control group.

**Conclusions:**

Our study indicates that patients who track their daily dietary habits using a mobile phone app are more likely to reach their nutritional goals than the control patients. Further large-scale studies are needed to confirm these initial findings and test the applicability on a broader basis.

## Introduction

The nutritional status of cancer patients has a significant influence on morbidity and mortality [1,2]. Cancer-induced inflammatory and catabolic processes lead to a progressive degradation of the muscle mass and the body’s fat deposits [3]. Up to 20% of cancer patients die as a result of weight loss and physical wasting [4]. In addition to tumor cachexia, loss of appetite, malabsorption and reduced nutrient uptake increase weight loss and further accelerate a decline in skeletal muscle mass [5]. Due to the anabolic resistance and increased turnover of muscle proteins, the need for high-quality proteins and amino acids is significantly higher in cancer patients [6]. An early start to nutritional therapy and stabilization of the body composition reduce morbidity and mortality in cancer patients [2]. According to national and international guidelines, screening of the nutritional status is recommended at the beginning of cancer-specific therapy as well as along the course of the disease [7,8]. Patients with suspected malnutrition should be given qualified nutritional therapeutic counseling to optimize their normal diet, and artificial nutrition should be provided if necessary [8]. Several studies have shown the benefits of nutritional care in cancer patients in relation to numerous aspects (malnutrition, quality of life, complications, mortality) [9-12].

The basis of any nutritional therapeutic concept is to record the individual nutrient intake of the patient. In this context, various dietary assessment methods are available. Retrospective methods have the disadvantage that their reliability strongly depends on the memory performance of patients (eg, 24-hour recall interviews, diet history interviews, or food frequency questionnaires). The gold standard of prospective nutritional protocols has long been the weighing protocol, but this is a burden for patients as all food must be weighed and recorded before being consumed. Therefore, prospective estimation records of food consumption are more patient-friendly. In Germany, for example, the Freiburg Diet Record (FB-DR) is one of the most commonly used estimation records, for which a computer-based evaluation procedure was developed (PRODI software, Nutri-Science GmbH). The database for the FB-DR is the Federal Food Key 3.02 developed by the German Federal Research Institute for Nutrition and Food. In the nutritional questionnaires of the FB-DR, patients record their diet on a standardized sheet. All estimation protocols have an acceptable expenditure of time because a tally list is used to note the food and drinks patients consumed during the course of the day. Qualified nutritionists use the records to determine the current energy and nutrient intake (micro- and macronutrients) of patients. This is important for individualized nutritional counseling and therapy. The food diaries are also used to detect preferences of patients for certain foods. However, none of the usual nutritional records has been developed specifically for cancer patients. Recording of the nutrient intake of oncologic patients would be very important, since dietary habits may change due to the specific disease situation and cancer therapy. National and international guidelines recommend a high-protein diet to counteract tumor cachexia and progressive muscle breakdown [8]. Due to issues relating to the disease and its therapy, appetite and taste disorders complicate the diet [13]. Thus, a special nutritional profile is typically present that must be individually tailored. However, in clinical routine, the resources for professional nutritional counseling for cancer patients are rather limited.

Mobile phone apps play an increasing role in the everyday use of electronic devices. Patient-specific data can be recorded and analyzed. These mobile phone apps are already being used successfully in other areas of nutrition consultation, and their benefit has been demonstrated in several studies [14-18].

However, in the field of oncology, comparable studies are rare and data about the usage of electronic aids are currently missing, especially the use of dedicated apps on mobile phones. Continuous monitoring of ingested nutrients and supporting individual nutritional goals by capturing individual dietary habits and dynamic changes during disease progression and tumor therapy are the target criteria of a mobile phone app for cancer patients. Therefore, the primary aim of this study was to evaluate the feasibility and applicability of a novel mobile phone app to assess and evaluate the nutritional status of patients with cancer diseases. Furthermore, we aimed at gaining first insights into whether the app may contribute to an improved nutritional status in cancer patients.

## Methods

### Patient Recruitment

Initially, 1400 nutritional records of 186 cancer patients from different oncology departments at University Hospital Erlangen were evaluated. The patients received nutritional care at the Hector Center for Nutrition, Exercise, and Sports in the Department of Internal Medicine 1 at the University Hospital Erlangen. For a detailed analysis of nutrient intake, an analysis of the FB-DR nutritional sheets was completed using PRODI version 6.2 organizational software for nutritional counseling and therapy (Nutri-Science GmbH). With the FB-DR, the energy and nutrient supply can be determined by using common kitchen dimensions. Including age, gender, and the number of documented days, a nutritional analysis was carried out using the DACH (Germany–Austria–Switzerland) reference values (German Society for Nutrition, Austrian Society for Nutrition, Swiss Society for Nutrition Research, Swiss Society for Nutrition). The analysis of the text logs was done externally in Java (Oracle Corporation) and Python (Python Software Foundation). The presence or risk of malnutrition was recorded using Nutritional Risk Screening 2002 [7,19-21].

The evaluation of the prospectively collected dietary charts allowed for optimizing the food selection and digital input of the daily diet into the app. The detailed recording of individual nutritional habits of cancer patients made it possible to detect foods missing from the FB-DR and preferences in food selection and develop the widest possible food choices for programming the app, based on the preferences of cancer patients. The nutritional goals were defined based on current national and international guideline recommendations on cancer nutrition [8].

**Figure 1:**
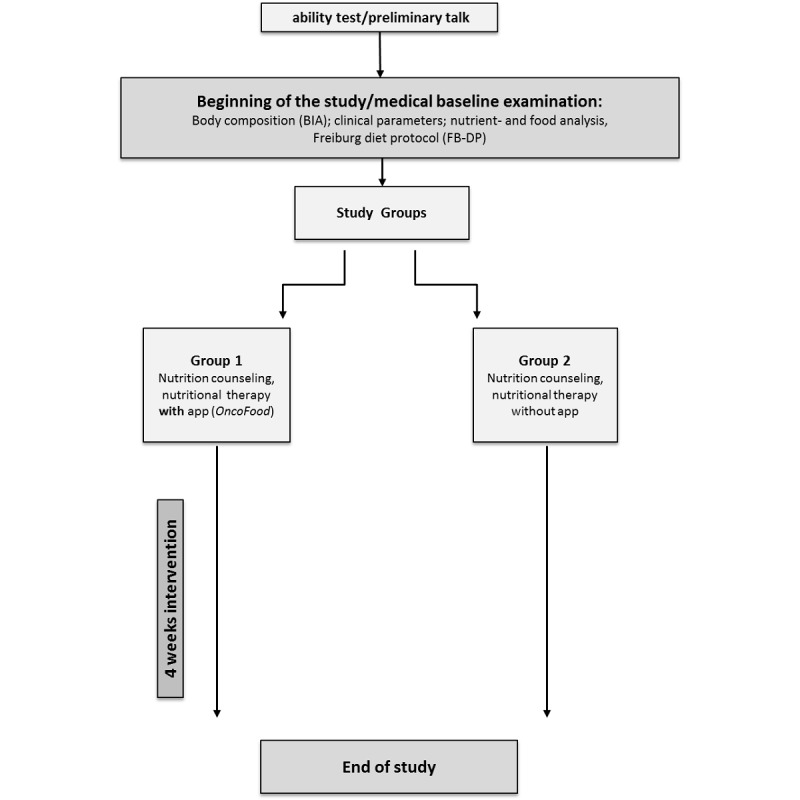
Design of study. BIA: bioimpedance analysis.

### Study Design

This study (see Figure 1 for flow diagram) recorded food intake over a 4-week period. After an initial nutritional analysis, both groups received professional nutritional advice with the aim to achieve the individually determined energy and nutrient requirements. Because of its explorative character, no explicit case number estimation was made.

In group 1 (app group), food intake was initially recorded using the FB-DR. In addition to the paper protocol, the participants were provided with a mobile phone with an Android operating system and the OncoFood app for nutritional documentation. To ensure that no entries were forgotten, the app reminded the user with an acoustic signal. The patient had the opportunity to check the current state and development of their energy and nutrient supply in the app.

In group 2 (usual care), food intake was recorded using a standardized paper record (FB-DR). Study participants completed the protocol for 3 days according to a given classification of the different foods. The nutritional analysis of the documented food was completed using PRODI software. The patients implemented the nutritional aims for the next 4 weeks by themselves.

### Setup of the App

OncoFood for Android mobile phones was developed in Java with Android Studio specifically for this study and programmed for the mobile phone Huawei Y6 (Huawei Technologies Co Ltd). Mobile phone selection was based on the average battery life and acquisition costs. Of course, the app can also be used on any other Android-based mobile phone. The app contains a database of more than 1300 nutrition facts for foods based on the German Nutrition Society’s nutritional value table [22]. The version used was updated based on the Federal Food Key 3.02.

Oncologic patients require high-calorie fluids, so 24 artificial, high-calorie liquids were added to the database with the appropriate nutritional values. Furthermore, foods listed by the cancer patients but missing from the top 200 foods in the FB-DR nutritional protocols in the preliminary evaluation were added. For each listed food, the water, energy, proteins, fat, carbohydrates, and fiber components were stored, which allowed a detailed nutritional calculation. Subjects were asked to enter the ingested foods and drinks into the app daily. The foods could either be entered by the screen keyboard or voice input. The last recorded foods could be saved and quickly revisited in a favorites list. Alternatively, the food could be selected from 16 categories such as fruit or bread spread. Compound and regularly scheduled meals (eg, spaghetti Bolognese, lasagna, or bread and butter) could be selected and saved for later reuse. The app reminded patients to enter their food intake every day at 9 am, 1 pm, and 7 pm. In case of a missed entry, the reminder function went off every 3 minutes (between the hours of 8 am and 10 pm only). Once a week, at 5 pm, patients were reminded with an acoustic signal to enter weight and appetite parameters collected to record the clinical status of the patient.

### Use of the App

**Figure 2:**
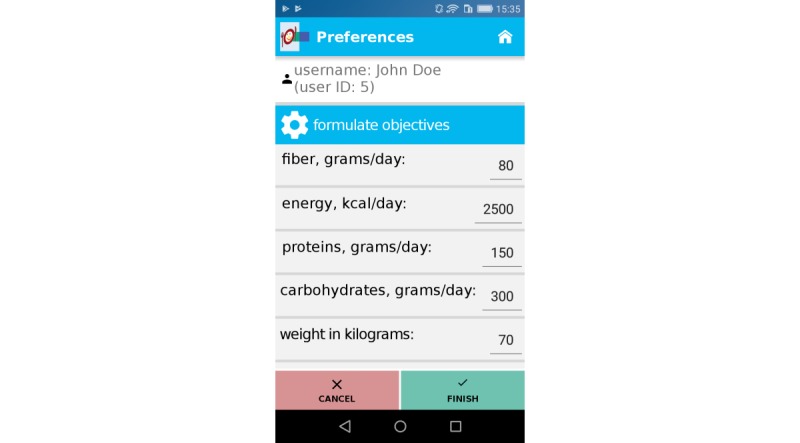
Nutritional goals and weight.

**Figure 3:**
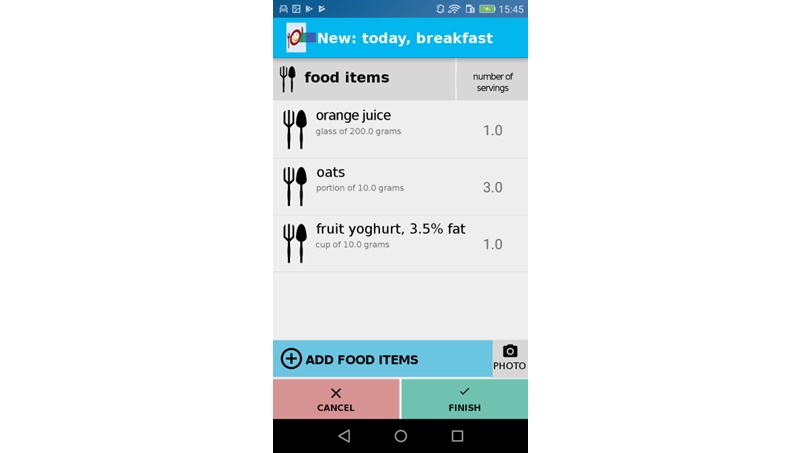
Daily input of consumed food.

**Figure 4:**
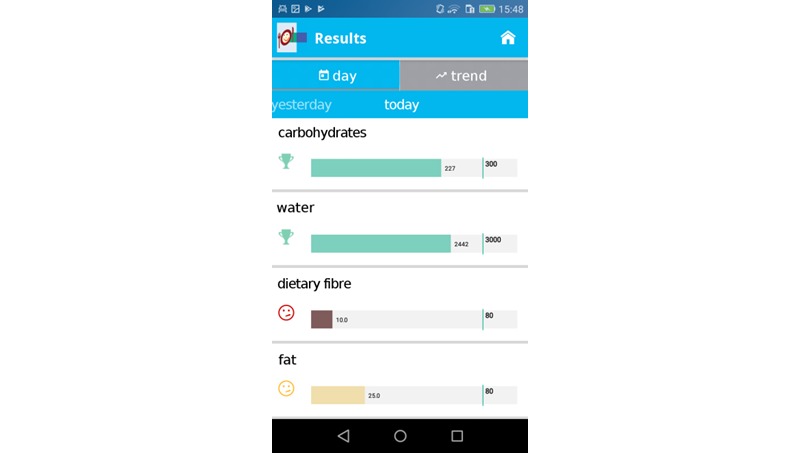
Record of daily nutritional goals met.

After individual nutritional status was recorded, OncoFood was configured individually for each patient by a physician and a nutritionist. For this purpose, nutritional goals and current weight were entered into the app (Figure 2). The mobile phone was then given to the patient. Patients entered consumed food daily; meals were stored separately and presented in an overview (Figure 3). Once the food was entered, charts showed whether patients reached their daily nutritional goals (Figure 4). The use of traffic light colors and symbol diagrams helped patients interpret what they had achieved. For example, a green cup represents the fact that a nutritional goal has been achieved with a nutrient. This aimed at motivating the patient to adhere to their nutritional plan.

### Bioimpedance Analysis

Bioimpedance analysis (BIA) is an easy-to-use, noninvasive method for determining the body composition of a patient. Each patient, regardless of group affiliation, received a measurement at the beginning and end of the study. The BIA is based on the measurement of body resistance against an electrical alternating current caused by the application of a voltage source. The BIA device used (Medical Body Composition Analyzer [mBCA], seca GmbH) is a multifrequency (5, 50, and 100 kHz) device with a hand/foot resistance of <300 ohm and a sandwich resistance of <30 ohm. Based on measured resistance and reactance values, the device calculates body cell mass and extracellular mass including intracellular water and extracellular water. In addition, the body fat mass is determined and the phase angle is calculated. The phase angle from the BIA measurement has been reviewed in several clinical trials and shown to be a useful prognostic marker for various diseases such as cirrhosis, HIV infection, and cancer [23]. With the modification of the phase angle to the standardized phase angle, its statement becomes more specific because it is related to the population (age, body mass index, nationality) [24]. The prognostic statement of the phase angle becomes more precise through the modification.

### Statistics

For categorical variables, numbers and percentages were determined and tested for baseline differences using the chi-square test. For continuous variables, averages and standard deviations were calculated at the beginning and end of the study in both groups (app subjects, controls). All the nutritional variables were given in the actual amount relative to the agreed target size. All continuous variables were tested with the Wilcoxon sign-rank test for differences within the groups before and after intervention. Differences in the final and starting values were calculated and tested using the Mann-Whitney *U* test. All analyses were performed with the statistical package R version 3.3.1 (R Foundation for Statistical Computing).

## Results

### Key Findings

Of the FB-DR protocols initially evaluated to interpret the dietary habits of cancer patients, 47.8% (89/186) were of women and 51.2% (97/186) were of men. Patients with different tumors (prostate, breast, esophagus, bronchi, stomach, pancreas, kidneys, liver, ovaries, colon, cecum, rectum, papillae, blood and hematopoietic system, lymphatic system, skin, or pleura) were included in the study. The mean age of the patients was 58.4 years (range 27-90 years). An average nutritional risk score of 3 points was determined.

**Figure 5:**
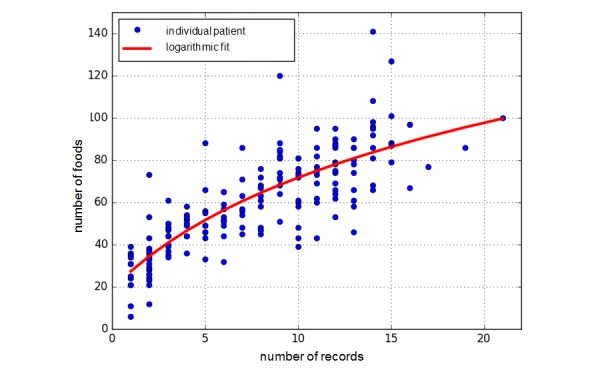
Increase in the number of different foods in terms of the number of protocols delivered.

Averages of 7.5 (SD 4.7) protocols per patient were completed. Although more than 7 protocols per patient were analyzed, no additional nutritional information could be evaluated. In particular, there were no differences in the number of specified foods recorded using the protocols (59.2 [SD 23.6]; Figure 5). Missing among the top 10 (Tables 1-3) foods most frequently mentioned in the FB-DR were beef and pork and their by-products. Drinks, fruit and fruit preparations, bread products, and dairy products were often consumed. Of the 172 foods listed in the FB-DR, 169 were recorded by the patients.

Out of the top 200 foods cited by our cancer patients as being preferred foods, 31 were missing from the FB-DR. Three foods (liquors, hamburgers, and cheeseburgers) that can be marked in the FB-DR were not even selected by oncologic patients (Table 4).

**Table 1:** Top 10 food choices documented by the participating cancer patients (excluding water and tea).

Position	Food	Terms (N)
1	Butter	155
2	White coffee	147
3	Coffee	136
4	Apple	126
5	Tomato	123
6	Milk, 1.5% fat	108
7	Banana	104
8	Espresso	94
9	Hen’s egg, cooked, with salt	83
10	Carrot	80

**Table 2:** Top 10 most consumed drinks documented by the cancer patients (including water and tea).

Position	Food	Terms (N)
1	Water	520
2	Herbal tea	266
3	Mineral water	206
4	White coffee	147
5	Coffee	136
6	Milk, 1.5% fat	108
7	Espresso	94
8	Coffee cream, 10% fat	63
9	Apple spritzer	59
10	Soft drinks	57

**Table 3:** Top 10 food choices documented by the cancer patients (excluding drinks).

Position	Food	Terms (N)
1	Butter	155
2	Apple	126
3	Tomato	123
4	Banana	104
5	Hen’s egg, cooked, with salt	83
6	Carrot	80
7	Cucumber	79
8	Rye whole grain bread	74
9	Jam	71
10	Red pepper	70

**Figure 6:**
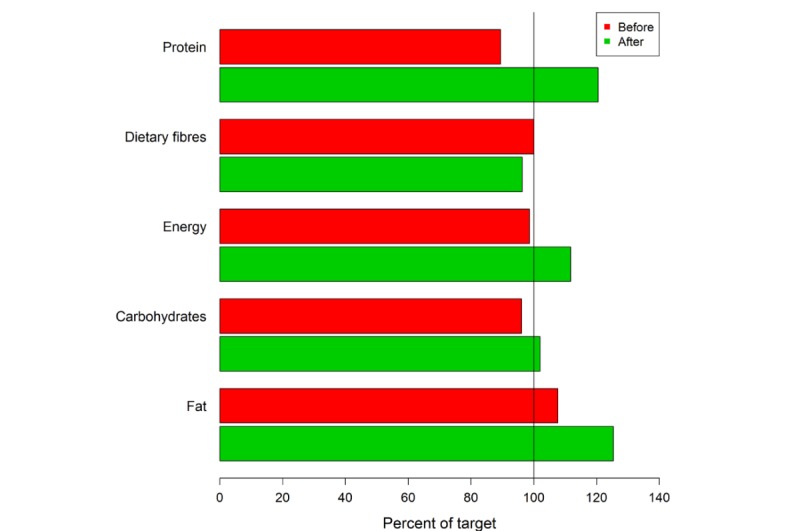
Four-week overview of nutritional goals reached by cancer patients using the app.

**Figure 7:**
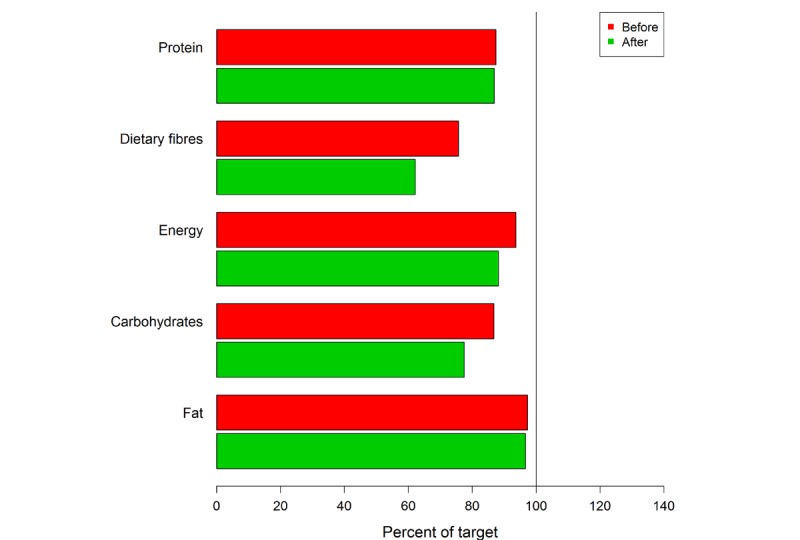
Four-week overview of nutritional goals reached by control group cancer patients.

**Figure 8:**
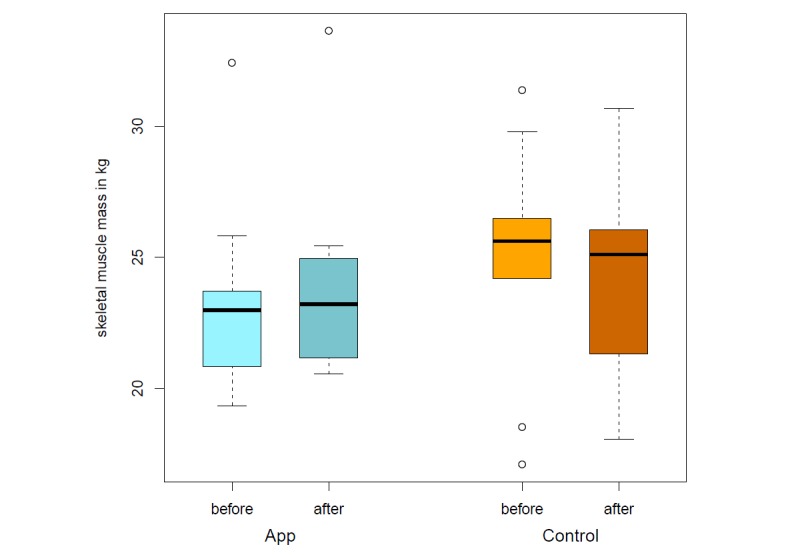
Skeletal muscle mass before and after intervention (app vs control).

**Figure 9:**
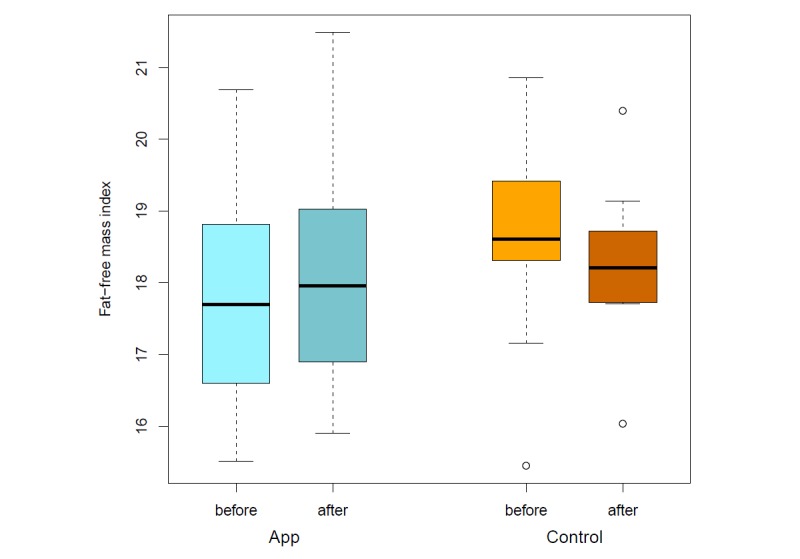
Fat-free mass index before and after intervention (app vs control).

**Figure 10:**
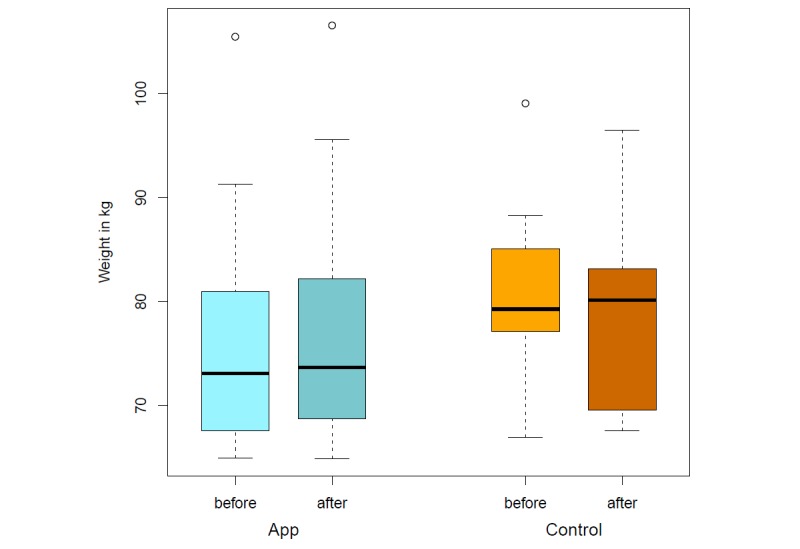
Weight before and after intervention (app vs control).

The app’s food database was programmed based on recorded nutrition protocols and the database of the German Nutrition Society [22]. Of the 39 patients (15 men and 24 women) originally recruited, 15 patients cancelled their participation before initiating the study. The most common reason for this was an inpatient admission. A total amount of 24 participants took part in the app study during the 4-week intervention. Twelve participants were assigned to a control group and 12 patients to the app group. This is still an appropriate sample size for this pilot investigation [25]. The group of patients suffering from a gastrointestinal tumor (n=16) was most frequently represented. Significant differences could be identified with regard to the achievement of the defined nutritional therapeutic goals. Thus, the protein and fat intake in the control group at the end of the study does not differ compared with the start of the study (*P*=.91). Fiber intake (*P*=.34), carbohydrates (*P*=.27), and total energy intake (*P*=.42) even show a worsening of the initial situation after 4 weeks. The patients who used OncoFood during the 4-week period achieved more than 100% of nutritional goals, especially with regard to protein and fat intake, as well as the total amount of energy (Figures 6 and 7) in comparison with the control cancer patients. They also achieved an adequate carbohydrate intake. The amount of fiber alone fell slightly compared with the previous value. The evaluation of the data shows a significant increase in skeletal muscle mass (*P*=.009; mean skeletal muscle mass 0.58 kg vs –0.61 kg; Figure 8) and fat-free mass (*P*=.03; Figure 9) for the app-using patients during the 4-week treatment. Weight gain and body mass index during the study period were significantly higher in the app subjects (*P*=.045; mean weight 1.03 kg vs –1.46 kg; Figure 10).

**Table 4:** Foods from the top 200 list from cancer patients that are not in the Freiburg Diet Record compared with foods from the Freiburg Diet Record that are not in the top 200 list of foods from cancer patients.

Position	Food
1	Liquor^a^
2	Hamburger^a^
3	Cheeseburger^a^
74	Formula diets
130	Pretzel
139	Vegetarian pastries
153	Gingerbread
158	Fried egg
159	Linseed
161	Malt beer
164	Doughnut
166	Soy milk
169	Avocado
170	Apple puree
172	Protein bread
173	Cheesecake
174	Mozzarella
175	Cappuccino
176	Lamb
177	Scrambled eggs
178	Tiramisu
182	Apple spritzer
183	Wheat bran
184	Espresso
185	Fruit salad
187	Feta cheese
190	Kefir
191	Goat cheese
192	Pita bread
193	Smoothie
194	Whole grain toast
195	Shandy
199	Tomato juice
200	Shrimp

^a^Foods in the Freiburg Diet Record that are not in the top 200 list.

### Challenges Using the App

The Android devices are owned by the clinic and were configured with a parental control app, allowing the subjects to access nothing but the OncoFood app and simplifying the task for them. Still, getting used to the new phone and app required effort and could be challenging for the individual subject. Nevertheless, the patients adapted easily to the phones and the OncoFood app running on them.

### Users’ Opinions

The patients in the group using OncoFood were asked to provide suggestions for improvement as well as positive and negative feedback. One particularly positive bit of feedback was that all meals in the database of the app were available. Also, the overview of the daily goal of nutrient intake was praised. Some noted that using the app would take too much time. One request was that the voice input of the app (via an activated internet connection) should also work offline. Additionally, many users also wished for recipe suggestions and the ability to make changes to existing and past data on foods and prepared meals.

## Discussion

### Principal Findings

On the basis of nutritional protocols, we were able to present for the first time that cancer patients not only show a changed diet but, in particular, have insufficient protein intake than is necessary for them. The analysis of standardized nutritional protocols showed that meat, for example, was not listed among the 25 most commonly consumed foods by tumor patients. Fish consumption was named last. However, not all of the preferred foods consumed by cancer patients were found in the standardized nutritional protocol. With adapted nutritional documentation and individualized nutritional care, the use of app assistance was associated with optimized nutritional status and could stabilize the body composition in cancer patients compared with conventional nutritional assistance.

Meat consumption has a higher priority in the healthy population compared with cancer patients. Meat is considered to be an important source of protein and contains a relevant amount of vitamins, trace elements, and minerals. A disturbed taste perception in cancer patients seems to be responsible for the avoidance of meat. In particular, the taste disturbance with the quality of bitter seems to increase the aversion to meat proteins [26]. This is especially unfavorable, as cancer patients require increased protein intake to counteract muscle breakdown caused by systemic inflammation and malignancy catabolic status.

A systematic recording of nutrition is the basis of nutritional treatment. We could show that standardized nutritional protocols such as the FB-DR may require special attention to specific dietary needs of specific disease populations. Among the 200 foods most commonly consumed by cancer patients, 31 foods were not included in the nutritional protocol. Artificial foods, such as enteral nutrition, were completely missing on the FB-DR. The results of our cancer database nutrition protocols served as a basis to optimize the food documentation of the OncoFood app.

The possibilities of nutritional counseling are often very limited in clinical practice due to the lack of sufficient consultation hours and human resources. In clinical routine, paper documentation is used to record the nutritional status, which is often completed retrospectively. This can lead to missing information about the daily food consumption.

A timely and detailed review of nutrient intake is very difficult to implement with the analogue protocol. In addition, the 1 to 3 day nutritional protocols do not provide complete records of the patient’s actual nutrition as a function of disease progression. In particular, tumor patients may have extremely high fluctuations in terms of nutrient uptake due to their tumor-specific therapy and the dynamic course of their disease. Not only the tumor cachexia but also the antitumor therapy (eg, chemotherapy, radiotherapy, or surgical therapy) influence food intake. A timely and continuous recording of these fluctuations in nutrient uptake by conventional nutritional protocols will hardly be possible.

So far, only Coughlin et al [27] have used a mobile phone app to record the nutritional status and activity levels in breast cancer patients. In this study, the participants were provided with an app that stores the information from 2 commercially available apps (Fitbit device for monitoring physical activity and the LoseIt! mobile phone app for monitoring and tracking diet and nutrition) and additionally contains information and tips for the prevention of breast cancer.

However, the patients did not initially receive professional dietary advice from dieticians who conducted an individual nutritional analysis and nutritional goals calculation as in our study. The data collection was based on the inputs of the patients in commercially available apps that were not specifically designed for cancer patients. Comparable to the OncoFood app used in our study, users were given a nutritional goal and a reminder to improve their compliance. In contrast to the OncoFood app, the option of a voice function was not available. A detailed analysis on which database the nutritional recommendation was based on was not reported. The use of apps in cancer patients has been studied mainly for the detection of cancer pain [28]. The benefits of the electronic input and regular monitoring of nutritional treatment were also reflected in our results. We could demonstrate that app users were able to achieve their previously defined nutritional therapeutic goals. The protein and fat intake as well as the total amount of energy were achieved more than 100%.

Compared with the app users, patients with conventional nutrition monitoring could not improve their nutritional status. In some cases a deterioration of the diet was recorded, so that the agreed nutritional goals could not be achieved. Thus, the protein and total energy intake of the control patients at the end of the study period was lower than at the beginning.

The skeletal muscle mass of patients with conventional nutritional intervention decreased over the 4-week study period (*P*=.06). In contrast, close monitoring of the nutritional status in the app group even resulted in significant weight gain (*P*=.05). In particular, skeletal muscle mass was significantly improved (*P*=.03).

Optimal management of nutritional therapy with significant improvement in body composition can otherwise only be shown with very close nutritional supervision. However, this proves to be difficult to perform in clinical routine. Time constraints, organizational circumstances, and socioeconomic aspects don’t allow an optimal and individual management of the patient. In this feasibility study, we could demonstrate that individually defined nutritional goals have a relevant influence on the eating behavior of the patients. Due to the easy handling and operation of the app, there was a high level of compliance and acceptance among our cancer patients. This allowed a fast and effective response to any changes in the nutritional needs of cancer patients. The considerable advantage of a mobile phone–controlled app has already been confirmed for other diseases, some of them lifestyle diseases such as diabetes mellitus, hypertension, and obesity [15,29-31].

For example, Ryan et al [15] developed a mobile phone app for people with type 1 diabetes. The aim was to positively influence glucose metabolism by giving patients the opportunity to incorporate the daily glucose measurements in an app and thus to provide a history overview.

There were also time advantages over traditional personal documentation by the patient. In the long term, the app users in this study showed an improvement in glucose parameters in type 1 diabetes patients. In contrast to our study, however, a nutritional recommendation based on type 1 diabetes was not offered.

Using new technology is one way to bridge the gap between what patients need and what health care can offer. This study evaluated a new digital health care platform. The use of a mobile phone app can be an effective and feasible method to improve the nutritional status of cancer patients.

### Limitations

Since the tumor collective in our study was very heterogeneous, it would be interesting to investigate the app for a uniform tumor disease. In addition, the 4-week study period can only provide an overview. Therefore, a longer observation period should be chosen in future studies. Prospectively, we want to shed light on the physical activity of tumor patients, so further studies are required.

### Conclusion

In accordance with national and international guidelines, cancer patients should follow a high-protein diet. We were able to show that closely guided nutrition therapy on a digital platform can not only improve the realization of the nutritional aims but also stabilize weight and skeletal muscle mass. The app was rated predominantly positively by the patients in terms of user satisfaction. Also, in relation to time and personnel costs, it offers advantages compared with traditional nutritional counseling and therapy. The app may be used in addition to conventional nutritional advice and therapy but also as a replacement for conventional therapy in every oncology patient. Further evaluation of the OncoFood app should be tested for validation in a larger collective of cancer patients.

## Abbreviations

BIA: bioimpedance analysis
FB-DR: Freiburg Diet Record

## References

[1] Sun J, Li W, Ke L, Tong Z, Ni H, Li G, Zhang L, Nie Y, Wang X, Ye X, Li N, Li J (2013). Early enteral nutrition prevents intra-abdominal hypertension and reduces the severity of severe acute pancreatitis compared with delayed enteral nutrition: a prospective pilot study. World J Surg.

[2] Paccagnella A, Morassutti I, Rosti G (2011). Nutritional intervention for improving treatment tolerance in cancer patients. Curr Opin Oncol.

[3] Tisdale MJ (2009). Mechanisms of cancer cachexia. Physiol Rev.

[4] Ryan AM, Power DG, Daly L, Cushen SJ, Ni Bhuachalla E, Prado CM (2016). Cancer-associated malnutrition, cachexia and sarcopenia: the skeleton in the hospital closet 40 years later. Proc Nutr Soc.

[5] O'Gorman P, McMillan DC, McArdle CS (1998). Impact of weight loss, appetite, and the inflammatory response on quality of life in gastrointestinal cancer patients. Nutr Cancer.

[6] van Dijk DP, van de Poll MC, Moses AG, Preston T, Olde DSW, Rensen SS, Deutz NE, Soeters PB, Ross JA, Fearon KC, Dejong CH (2015). Effects of oral meal feeding on whole body protein breakdown and protein synthesis in cachectic pancreatic cancer patients. J Cachexia Sarcopenia Muscle.

[7] Kondrup J, Rasmussen HH, Hamberg O, Stanga Z, Ad Hoc ESPEN Working Group (2003). Nutritional risk screening (NRS 2002): a new method based on an analysis of controlled clinical trials. Clin Nutr.

[8] Arends J, Bachmann P, Baracos V, Barthelemy N, Bertz H, Bozzetti F, Fearon K, Hütterer E, Isenring E, Kaasa S, Krznaric Z, Laird B, Larsson M, Laviano A, Mühlebach S, Muscaritoli M, Oldervoll L, Ravasco P, Solheim T, Strasser F, Preiser J (2017). ESPEN guidelines on nutrition in cancer patients. Clin Nutr.

[9] Silander E, Nyman J, Hammerlid E (2013). An exploration of factors predicting malnutrition in patients with advanced head and neck cancer. Laryngoscope.

[10] Morton RP, Crowder VL, Mawdsley R, Ong E, Izzard M (2009). Elective gastrostomy, nutritional status and quality of life in advanced head and neck cancer patients receiving chemoradiotherapy. ANZ J Surg.

[11] Locher JL, Bonner JA, Carroll WR, Caudell JJ, Keith JN, Kilgore ML, Ritchie CS, Roth DL, Tajeu GS, Allison JJ (2011). Prophylactic percutaneous endoscopic gastrostomy tube placement in treatment of head and neck cancer: a comprehensive review and call for evidence-based medicine. JPEN J Parenter Enteral Nutr.

[12] Richards DM, Tanikella R, Arora G, Guha S, Dekovich AA (2013). Percutaneous endoscopic gastrostomy in cancer patients: predictors of 30-day complications, 30-day mortality, and overall mortality. Dig Dis Sci.

[13] Gamper E, Giesinger JM, Oberguggenberger A, Kemmler G, Wintner LM, Gattringer K, Sperner-Unterweger B, Holzner B, Zabernigg A (2012). Taste alterations in breast and gynaecological cancer patients receiving chemotherapy: prevalence, course of severity, and quality of life correlates. Acta Oncol.

[14] Burke LE, Conroy MB, Sereika SM, Elci OU, Styn MA, Acharya SD, Sevick MA, Ewing LJ, Glanz K (2011). The effect of electronic self-monitoring on weight loss and dietary intake: a randomized behavioral weight loss trial. Obesity (Silver Spring).

[15] Ryan EA, Holland J, Stroulia E, Bazelli B, Babwik SA, Li H, Senior P, Greiner R (2017). Improved A1C levels in type 1 diabetes with smartphone app use. Can J Diabetes.

[16] Garnett C, Crane D, West R, Michie S, Brown J, Winstock A (2017). User characteristics of a smartphone app to reduce alcohol consumption. Transl Behav Med.

[17] Jimoh F, Lund EK, Harvey LJ, Frost C, Lay WJ, Roe MA, Berry R, Finglas PM (2018). Comparing diet and exercise monitoring using smartphone app and paper diary: a two-phase intervention study. JMIR Mhealth Uhealth.

[18] Gabrielli S, Dianti M, Maimone R, Betta M, Filippi L, Ghezzi M, Forti S (2017). Design of a mobile app for nutrition education (trec-lifestyle) and formative evaluation with families of overweight children. JMIR Mhealth Uhealth.

[19] Ockenga J, Valentini L (2005). Review article: anorexia and cachexia in gastrointestinal cancer. Aliment Pharmacol Ther.

[20] Mercadal-Orfila G, Lluch-Taltavull J, Campillo-Artero C, Torrent-Quetglas M (2012). Association between nutritional risk based on the NRS-2002 test and hospital morbidity and mortality. Nutr Hosp.

[21] Orell-Kotikangas H, Österlund P, Saarilahti K, Ravasco P, Schwab U, Mäkitie AA (2015). NRS-2002 for pre-treatment nutritional risk screening and nutritional status assessment in head and neck cancer patients. Support Care Cancer.

[22] Heseker H, Heseker B (2017). Die Nährwerttabelle 2016.

[23] Norman K, Stobäus N, Pirlich M, Bosy-Westphal A (2012). Bioelectrical phase angle and impedance vector analysis—clinical relevance and applicability of impedance parameters. Clin Nutr.

[24] Bosy-Westphal A, Danielzik S, Dörhöfer R, Later W, Wiese S, Müller MJ (2006). Phase angle from bioelectrical impedance analysis: population reference values by age, sex, and body mass index. JPEN J Parenter Enteral Nutr.

[25] Julious SA (2005). Sample size of 12 per group rule of thumb for a pilot study. Pharmaceut. Statist.

[26] Schalk P, Kohl M, Herrmann HJ, Schwappacher R, Rimmele ME, Buettner A, Siebler J, Neurath MF, Zopf Y (2018). Influence of cancer and acute inflammatory disease on taste perception: a clinical pilot study. Support Care Cancer.

[27] Coughlin SS, Besenyi GM, Bowen D, De Leo G (2017). Development of the Physical activity and Your Nutrition for Cancer (PYNC) smartphone app for preventing breast cancer in women. Mhealth.

[28] Jibb LA, Stevens B, Nathan PC, Seto E, Cafazzo J, Johnston D, Hum V, Stinson J (2017). Implementation and preliminary effectiveness of a real-time pain management smartphone app for adolescents with cancer: a multicenter pilot clinical study. Pediatr Blood Cancer.

[29] Finkelstein J, Bedra M, Li X, Wood J, Ouyang P (2015). Mobile app to reduce inactivity in sedentary overweight women. Stud Health Technol Inform.

[30] Johnston N, Bodegard J, Jerström S, Åkesson J, Brorsson H, Alfredsson J, Albertsson PA, Karlsson J, Varenhorst C (2016). Effects of interactive patient smartphone support app on drug adherence and lifestyle changes in myocardial infarction patients: a randomized study. Am Heart J.

[31] Kang H, Park H (2016). A mobile app for hypertension management based on clinical practice guidelines: development and deployment. JMIR Mhealth Uhealth.

